# Exaggerated evolution of male armaments via male–male competition

**DOI:** 10.1002/ece3.7546

**Published:** 2021-05-02

**Authors:** Maica Krizna D. Areja‐Gavina, Monica C. Torres, Gimelle B. Gamilla, Tomohiko Sakaguchi, Hiromu Ito, Jomar F. Rabajante, Jerrold M. Tubay, Jin Yoshimura, Satoru Morita

**Affiliations:** ^1^ Mathematics Division Institute of Mathematical Sciences and Physics University of the Philippines Los Baños College Philippines; ^2^ Department of Mathematical and Systems Engineering Shizuoka University Hamamatsu Japan; ^3^ Graduate School of Science and Technology Shizuoka University Hamamatsu Japan; ^4^ Department of International Health Institute of Tropical Medicine Nagasaki University Nagasaki Japan; ^5^ Department of General Systems Studies University of Tokyo Tokyo Japan; ^6^ Faculty of Education University of the Philippines Open University College Laguna Philippines; ^7^ Marine Biosystems Research Center Chiba University Uchiura Japan; ^8^ Department of Environmental and Forest Biology State University of New York College of Environmental Science and Forestry Syracuse NY USA

**Keywords:** armament, evolution, game theory, male competition

## Abstract

Males usually compete to gain access to prospective mates. Through this male–male competition, superior males have a higher chance of passing on their traits to the next generation of male offspring. One category of male traits is armaments, which are weapons used during competition, for example, the chelae of fiddler crabs and the antlers of deer. One consequence of intrasexual selection is the exaggerated evolution of armaments, which can be limited by trade‐offs, such as trade‐offs with male body size. Here, we formulate a game‐theoretic sexual selection model to explore the exaggerated evolution of armaments through male–male competition. The model is used to determine how competition affects the evolution of an armament that is subject to trade‐offs. Our simulation can be used to support the exaggerated evolution hypothesis, that is, male–male competition escalates the rate of evolution of armaments.

## Graphical abstract

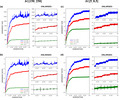

## 1 INTRODUCTION

The exaggerated evolution of secondary sexual traits, such as armaments, may be attributed to mate selection in males (Darwin, 1871; Diaz‐Munoz et al., 2014; Lorenz, 2002; Yoshimura, 1992). Many evolutionary studies have concentrated on male ornaments with exaggerated features, such as the tail of a peacock, whereas fewer have focused on male armaments (Berglund et al., 1996; Johnstone et al., 2009; McGhee et al., 2007; Yoshimura, 1992). Males with exaggerated sexual traits often obtain more mates than those that have less exaggerated sexual traits (Yoshimura, 1992). The main function of exaggerated ornaments is to increase the genetic contribution of the male to the next generation through increased sexual attractiveness (Berglund et al., 1996). However, some armaments can also be used as signals that inform other males regarding the fighting prowess or superiority of the bearer and thus affect the genetic contribution to the next generation through aggressive competition (Berglund et al., 1996). Armaments, such as the chelae of fiddler crabs and the antlers of deers, can be better preserved by not engaging in a fight in cases where the male is likely to lose the fight. Some animals concede in competition not only to prevent damage to their armaments but also to avoid the energetic cost of fighting and associated injuries, which are sometimes lethal (Arnott & Elwood, 2009).

In a male contest, the male visually assesses the strength of his rival to reduce fighting time and cost (Eberhard et al., 2018; McCullough et al., 2016). Through ritualistic display, rivals can reduce fighting costs considerably (Eberhard et al., 2018; Enquist & Leimar, 1983; Lorenz, 1963). Some animal species have ritualistic fighting behaviors such as posturing, maintaining eye contact, roaring, and engaging in pushing to deter their rivals (Lorenz, 2002). Armament size can be used as a proximate cue to the size and strength of an opponent in his decision as to whether he should retreat or continue fighting. Males of polygynous species are well known to engage in dangerous competitions (Weckerly, 1998; Yoshimura, 1992). Males of inferior fighting ability either die out or invest in fighting‐related traits. Take into consideration that maintaining superior fighting ability can be costly, especially when it deteriorates rapidly while other surviving males become increasingly competitive (Clutton‐Brock & Huchard, 2013).

In male**–**male competition, typical traits associated with fighting ability are body size and armament size. Studies show that there is an allometric relationship between body size and armament size (Bonduriansky, 2007; Kodrid‐Brown et al., 2006; Tidiere et al., 2017). Positive allometry of secondary sexual traits of males is linked to its function as a weapon, such as antlers of deers, horns of beetles, and the chelae of fiddler crabs (Eberhard et al., 2018; Lincoln, 1994). Due to natural selection, a large armament is beneficial to some extent but is detrimental to survival once it exceeds a threshold size (Lincoln, 1994). A large body size, together with musculature and aggressive behavior, is often necessary for success in a competition (Lincoln, 1994). However, if body size is equal between rivals, the male with remarkable armament is more likely to win the fight. Two males of equal body size may differ in armament size due to the proportions of the traits of male and female parents that are inherited by the offspring, mutation of the traits in offsprings, and the variation in resource allocation during development. A larger armament suggests a greater possibility of winning a mate competition (Lincoln, 1994). Hence, an optimal strategy for a male of the same body size as his rival but with a significantly smaller armament is to retreat from a fight to minimize damage. However, large body size and large armament size do not guarantee victory in a competition, as the fighting ability is also affected by physical condition. Here, the physical condition refers to the physical strength of a male, which ranges from 0 (dead) to 100 (strongest). Physical condition deteriorates as an individual engages in an increasing number of fights since physical fight results in injury to both competitors. Also, mortality increases as the size of the armament approaches the size of the body (Tidiere et al., 2017).

In this paper, we use a game‐theoretic sexual selection model to characterize the exaggerated evolution hypothesis, that is, male–male competition escalates the rate of evolution of armaments. To test the hypothesis, we use numerical simulation to investigate the factors that affect the evolution of armaments. The following assumptions are invoked in the model: (a) Fighting ability is dependent on body size and armament size, (b) mortality rate is affected by natural death, and the relationship between body size and armament size, and (c) there is a phenotypic correlation between a fitness sensitive trait and a secondary sexual trait (e.g., allometry of body size and armament size). To simplify the model in order to focus on the exaggerated evolution hypothesis, we assume that the males are incapable of replenishing their energetic pool by acquiring more resources after a fight, and the preference of the females is not considered in the selection process of mates. Using the model, we show that male–male competition may result in the nonadaptive by‐product evolution of armament size and ratio in animals. In addition, we assume that individuals with superior fighting ability have a higher probability of winning a competition. Superior fighting ability means having a large body size, remarkable armament, and great physical condition. A male that retains superior fighting ability after engaging in numerous competitions wins one or more mates.

## 2 MODEL AND RESULTS

We consider *n*_1_ males that compete to mate with *n*_2_ sexually matured females at generation *t*. The winner is dictated by the fighting ability of each male, which is a function of the armament size, body size, and physical condition of the male. The winner will then have the opportunity to mate and reproduce. We denote the armament size of males who join the competition at generation *t* as *a_t_*. The body size *b* is normally distributed with mean *µ* and standard deviation *σ*, that is, *b* ~ *N*(*µ*,*σ* ^2^) and ranges from 150 to 250 (Castelló et al., 2016). Each male has an initial physical condition *f_i_*(1)=100, which reduces after engaging in a fight.

Two superior males in terms of armament size may engage in a fight. The parameter *r_at_* is equal to the size of the large armament divided by the size of the smaller one. Before each competition begins, the armament sizes of males are compared using the following rules: (a) If the ratio between the armaments of the competing individuals, *r_at_*, is greater than or equal to the threshold ratio *r_thres_* (≥1), then the winner by default (without physical competition between the rivals) is the male with the larger armament (Karino et al., 2005), and (b) if the ratio between two armaments, *r_at_*, is less than *r_thres_*, then the male with superior fighting ability is declared the winner after the physical competition. This means for ratios below the threshold, fight outcomes are determined entirely by the body size, armament size, and the physical condition of the animal. The first criterion is assumed because it is expected that males with relatively small armaments retreat from a fight to avoid extensive physical damage or death. For case (ii), the fighting ability of individual *i* for his next fight (i.e., fight *j + *1) is computed using the following equation:

(1) $$f_{i}\left(j+1\right)=\overset{armament\;size}{\overbrace{a_{t}}}\times \overset{power\;functionofbody\;size}{\overbrace{b^{k}}}\times \overset{physical\;condition}{\overbrace{\frac{f_{i}(j)‐cost}{100}}},\forall i$$

where *f_i_*(*j*) is the fighting ability of an individual during fight *j*.

A physical fight resulting in injury to both competitors is represented by a cost. The cost to each competitor depends on the outcome of the fight, that is, the winner incurs a cost of *W,* while the loser incurs a cost of *L* (i.e., *L* > *W*), which is deducted from the physical condition of each competitor. The winner of the competition will then have the chance to reproduce and pass its traits to its offspring. Note that the cost we consider here is couched specifically as an injury, which means a fight between two small individuals will not result in a high probability of injury, whereas larger and stronger individuals would be more likely to injure each other.

**FIGURE 1:**
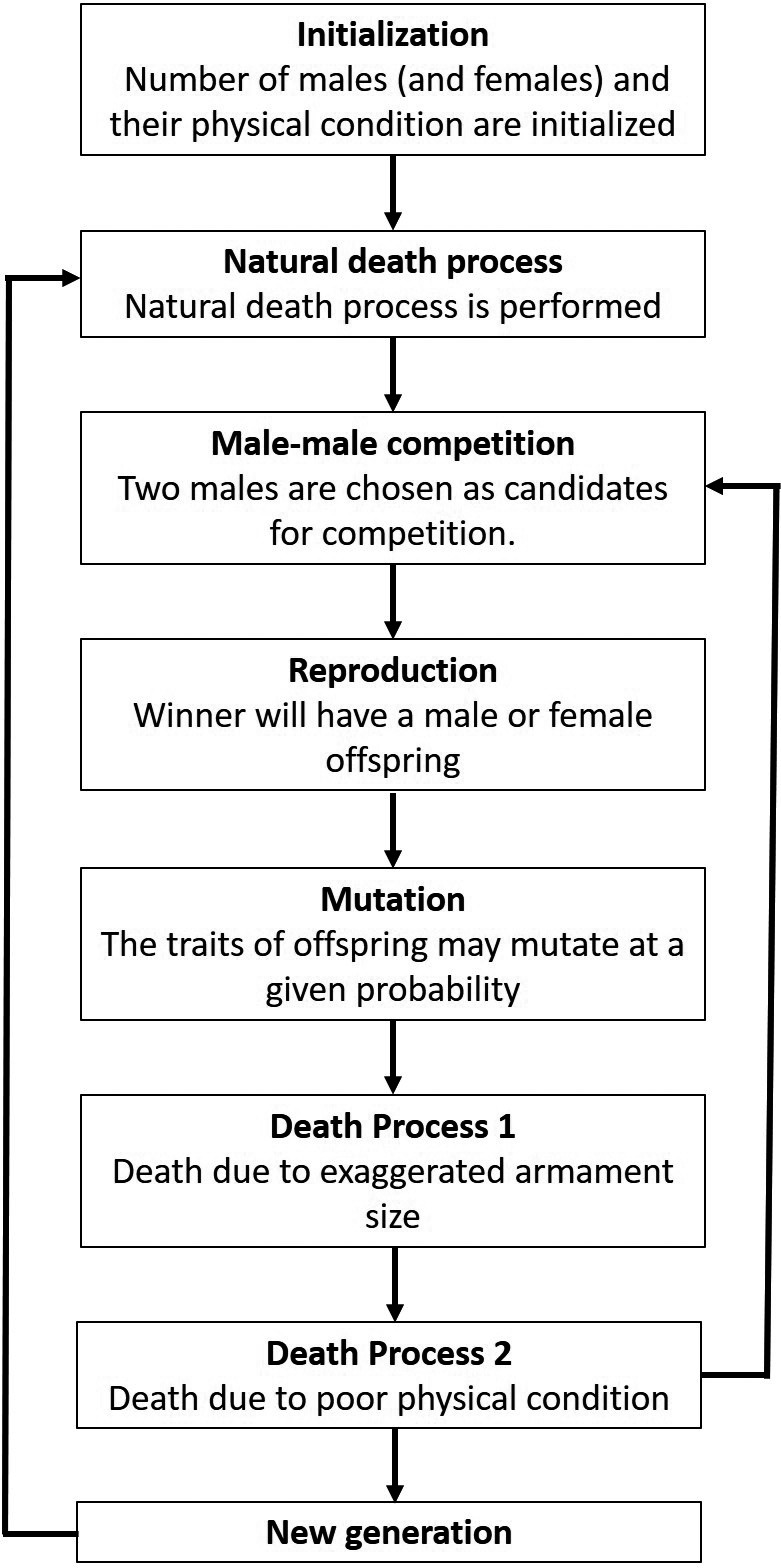
Schematic diagram of the evolution of armament size via male competition during mating, and the effect of armament ratio on mortality rate. Diagram showing the steps of the simulation. In a simulation run, fights among males are repeatedly executed per generation. Then for every fight, the winners will mate the randomly chosen female. After that, the females will give birth, until all the individuals who die in the previous generation are replaced. This was carried out for *N* generations

Let *x*, *y* be the genotypic traits of male and female parents. The proportions of the genotypic traits of male and female parents that are inherited by the offspring are represented by *P*_1_ and *P*_2_, respectively, where *P*_1_+*P*_2_=1. Here, we define *P*_1_ *x*+*P*_2_ *y* as the average genotypic traits from the parents inherited by the offspring. The initial values for *P*_1_ and *P*_2_ are set randomly. Moreover, the traits of offspring mutate at a certain probability *M*. Also, evolutionary allometry between armament size *a_t_* and body size *b* of males is nonlinear within each group. The allometric relationship between armament size at generation *t* and body size *b* can be described as follows (Bonduriansky, 2007; Eberhard et al., 2018; Kodrid‐Brown et al., 2006; Lincoln, 1992, 1994; Yoshimura, 1992):

(2) $$a_{t}=b^{k}\left(P_{1}x+P_{2}y\right)$$

where *k* is the allometric slope. According to Kodrid‐Brown et al. (2006), the slopes for male armaments range from 0.93 to 15.7. The armament size of the offspring at generation *t* is shown in the schematic diagram in Figure 1. We denote *P*_1_ *x*+*P*_2_ *y* by the ratio *a_rt_*, that is,

(3) $$a_{\mathrm{rt}}=\frac{a_{t}}{b^{k}}=P_{1}x+P_{2}y.$$

In a simulation run, fights are repeatedly executed per generation. Then for every fight, the winners will mate the randomly chosen female. This means that one female can mate only with the winning male for every fight. After that, the female will give birth, until all the individuals who die in the previous generation are replaced. This process is repeated for *N* generations to determine the evolution of armament size (*a_t_*) and the ratio of armament size to body size (*a_rt_*). Note that for each generation, only the surviving males from the previous generation will engage in the mate competition. Moreover, the model parameters in the simulation run were estimated based on existing studies of male deer (Table 1).

**TABLE 1:** Description and default values of the parameters

Parameter	Description	Default value	Reference
*n_1_*	Initial number of males	100	Assigned
*n_2_*	Initial number of females	100	Assigned
*P*_1_ *x*	Initial contribution of the male trait to the offspring	0.01*rand{0,1}	Assigned
*P*_2_ *y*	Initial contribution of the female trait to the offspring	0.01*rand{0,1}	Assigned
*M*	Probability of mutation	0.00001	Assigned
*N*	Number of generations	100,000	Assigned
*d*	Natural death rate	0.31	Sönnichsen et al. (2017)
*k*	Allometric slope	1.35	Ungerfeld et al. (2011), Geist (1998)
*b*	Body size	[150,250]	Castello (2016)
*a_rt_*	Armament ratio (adult males, 5–6 years old)	8.7 ± 0.4	Ungerfeld et al. (2011)

Note

The model parameters were estimated based on existing studies of male deer.

For each generation, excluding the new births, individuals die at a natural death rate d. Natural death is not associated with male–male competition and the development of exaggerated armament size, for example, death due to diseases, destruction of habitat, or natural disasters. As an effect of competitive superiority, it is expected that armament size *a_t_* will increase rapidly with respect to body size *b*. To prevent boundless exaggeration of armament size, a large mortality rate is assigned to individuals with an armament size larger than their body size (i.e., *a_rt_* >1). An example of an insect where its weaponry is larger than its body size is the harlequin beetle (*Acrocinus longimanus*). Individuals of body size less than or equal to their armament size are assigned a mortality rate of zero. The general form of the mortality rate function is given by (Ditchkoff et al., 2001; Johnstone et al., 2009).

(4) $$m\left(a_{rt}\right)=\left\{\begin{array}{cc} 0 & ,\;a_{rt}\leq 1\\ \frac{1}{{\left(\beta ‐1\right)}^{\alpha }}{\left(a_{rt}‐1\right)}^{\alpha } & ,\;a_{rt}>1 \end{array}\right)$$

where β and α are the values of *a_rt_* when *m*(*a_rt_*)=1 and a chosen exponent, respectively. The rationale for imposing a high mortality rate is the positive evolutionary allometric relationship between body size and armament size, as natural selection does not favor armaments that are larger than the body. Thus, the model is affected not only by sexual selection but also by natural selection.

**FIGURE 2:**
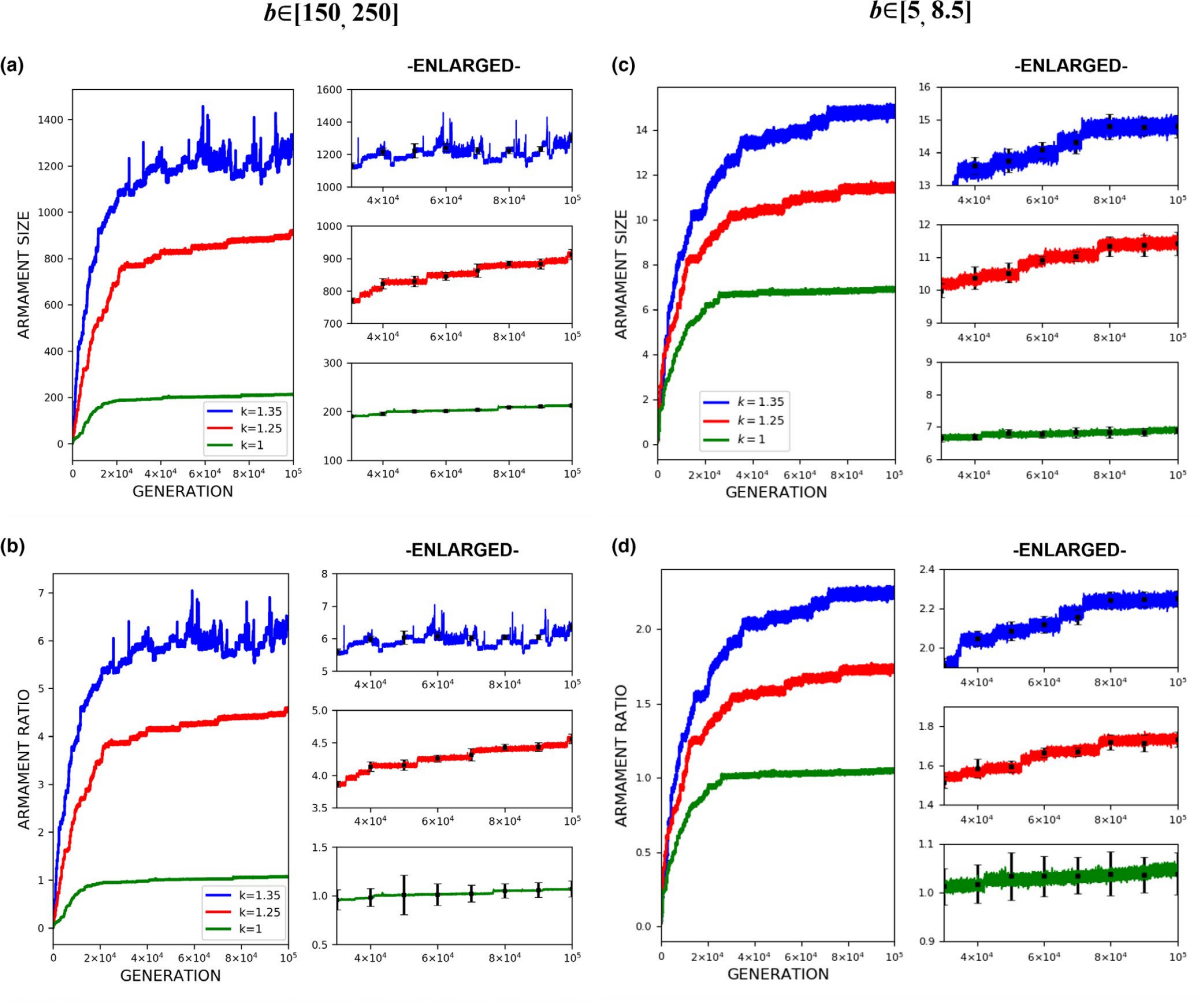
Evolution of armament size and ratio for each generation *t* as influenced by allometric slope *k*. Enlarged diagrams show the evolution of (a, c) armament size and (b, d) armament ratio for generations *t*∈ [40000,100000]. The nonlinearity of body size was set as follows: *k* = 1.35 (blue), *k* = 1.25 (red), and *k* = 1 (green). Parameter values (see Table 1): *n*_1_=100, *n*_2_=100, *N* = 100,000, *f _i_*(1)=100, (a‐b) larger body size, *b* ~ *N*(200,15), where *b*∈ [*b_min_*=150_,_ *b_max_*=250], (c‐d) smaller body size, *b* ~ *N*(6,15), where *b*∈ [*b_min_*=5_,_ *b_max_*=8.5], *r_thres_*=1.5, *W* = 5, *L* = 10, and *m*(*a_rt_*)= $\frac{1}{9^{10}}{\left(a_{\mathrm{rt}}‐1\right)}^{10}$. For our simulation analysis, we used the package Spyder (Python 3.7). Average and standard deviation of 20 runs for 100,000 generations are shown

**FIGURE 3:**
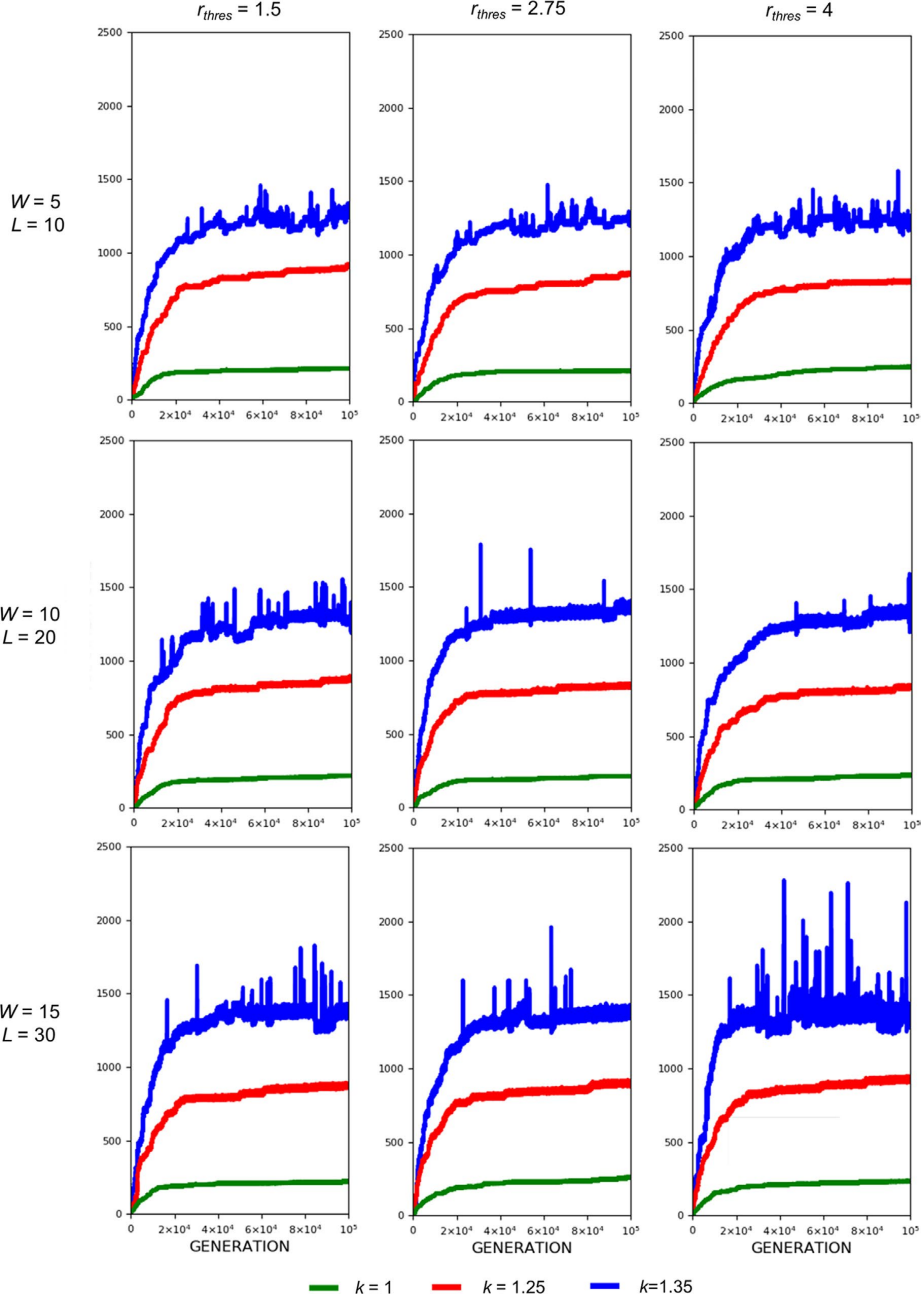
Evolution of armament size for each generation *t* as influenced by allometric slope *k*. The nonlinearity of body size was set as follows: *k* = 1.35 (blue), *k* = 1.25 (red), and *k* = 1 (green). Parameter values (see Table 1): *n*_1_=100, *n*_2_=100, *N* = 100,000, *f _i_*(1)=100, *b* ~ *N*(200,15), where *b*∈ [*b_min_*=150_,_ *b_max_*=250], *r_thres_*∈{1.5,2.75,4}, *W*∈{5,10,15}, *L*∈{10,20,30}, and *m*(*a_rt_*)= $\frac{1}{9^{10}}{\left(a_{\mathrm{rt}}‐1\right)}^{10}$. For our simulation analysis, we used the package Spyder (Python 3.7). Average of 10 runs for 100,000 generations is shown

**FIGURE 4:**
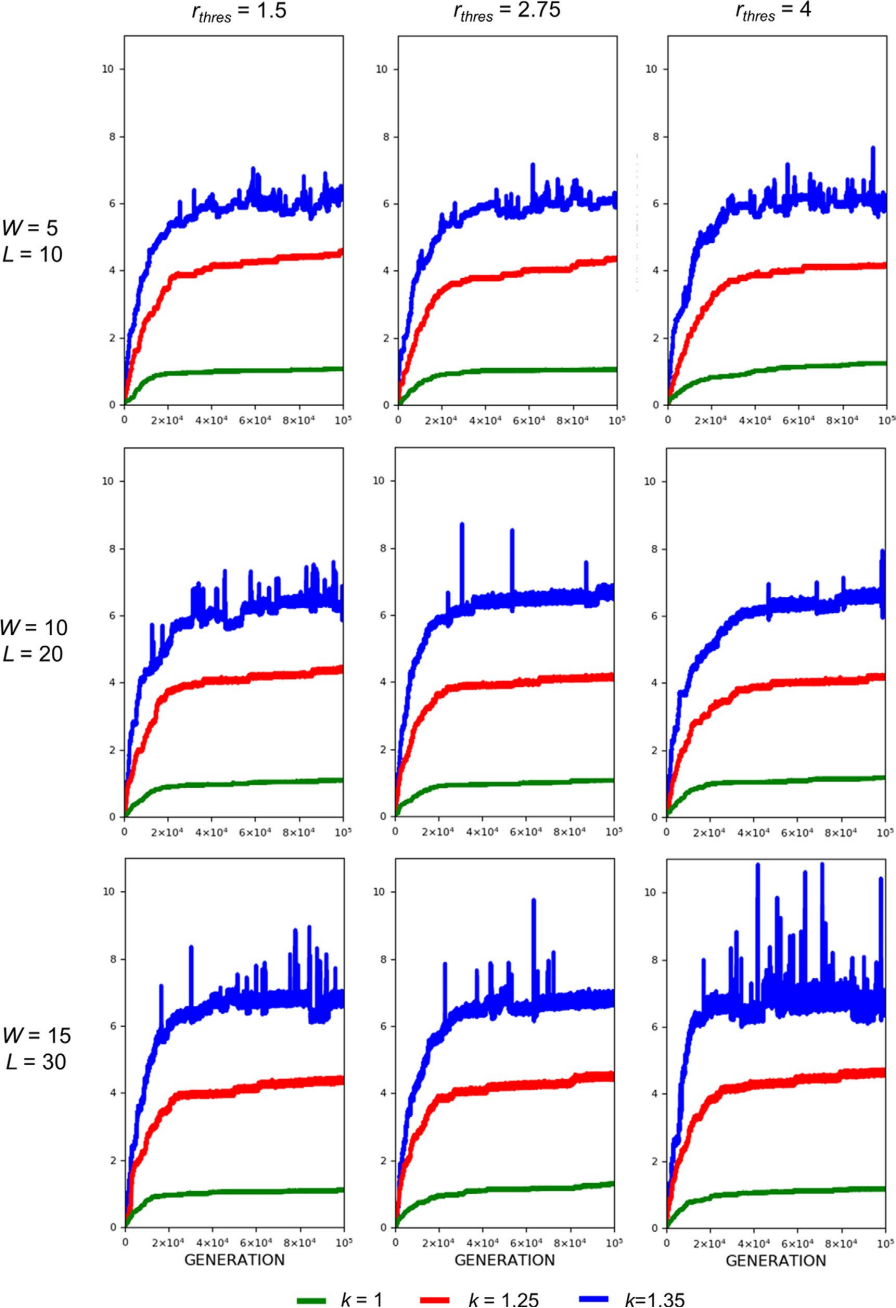
Evolution of armament ratio for each generation *t* as influenced by allometric slope *k*. The nonlinearity of body size was set as follows: *k* = 1.35 (blue), *k* = 1.25 (red), and *k* = 1 (green). Parameter values (see Table 1): *n*_1_=100, *n*_2_=100, *N* = 100,000, *f _i_*(1)=100, *b* ~ *N*(200,15), where *b*∈ [*b_min_*=150_,_ *b_max_*=250], *r_thres_*∈{1.5,2.75,4}, *W*∈{5,10,15}, *L*∈{10,20,30}, and *m*(*a_rt_*)= $\frac{1}{9^{10}}{\left(a_{\mathrm{rt}}‐1\right)}^{10}$. For our simulation analysis, we used the package Spyder (Python 3.7). Average of 10 runs for 100,000 generations is shown

**FIGURE 5:**
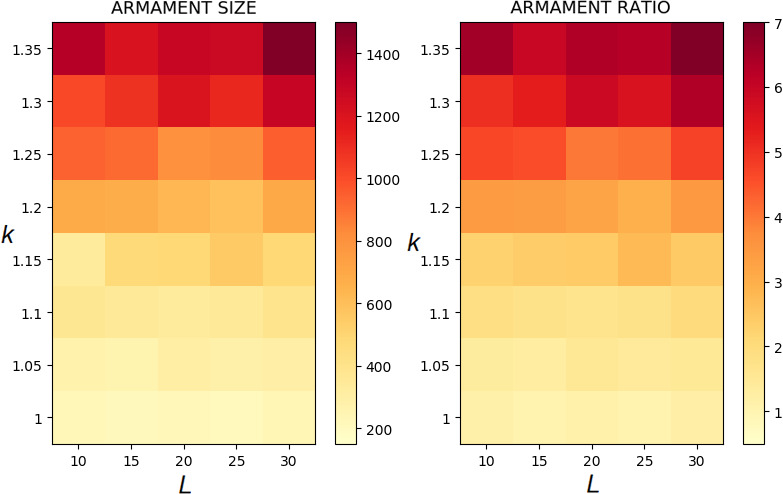
The effect of competition cost and allometric ratio to the size and ratio of the armament. Parameters (see Table 1): *n*_1_=100, *n*_2_=100, *b* ~ *N*(200,15), where *b*∈ [*b_min_*=150_,_ *b_max_*=250], *N* = 100,000, *r_thres_*=1.5, *W* = 5, and *m*(*a_rt_*)= $\frac{1}{9^{10}}{\left(a_{\mathrm{rt}}‐1\right)}^{10}$. For our simulation analysis, we used the package Spyder (Python 3.7). Average of 10 runs for 100,000 generations is shown

The evolution of exaggerated armament size occurs when the fighting ability of a male is affected by the allometric slope. Figures 2, 3, 4 show that the armament size and armament ratio are influenced by the allometric slope. The evolution of exaggerated armament sizes is more pronounced when allometric slope is greater than 1(Figure 2). This can also be observed in any combination of competition costs (W and L) and *r_thres_* explored in this study (Figures 3 and 4). Moreover, this result can also be seen at different ratios of competition costs (Figure 5). In addition, the simulation model presented here can describe the evolution of armament size of animals with different body size supported by Figure 2. Here, we have shown the robustness of predictions of the proposed model with respect to body size. We observed that the evolutionary dynamics of armament size are similar for any body size.

**FIGURE 6:**
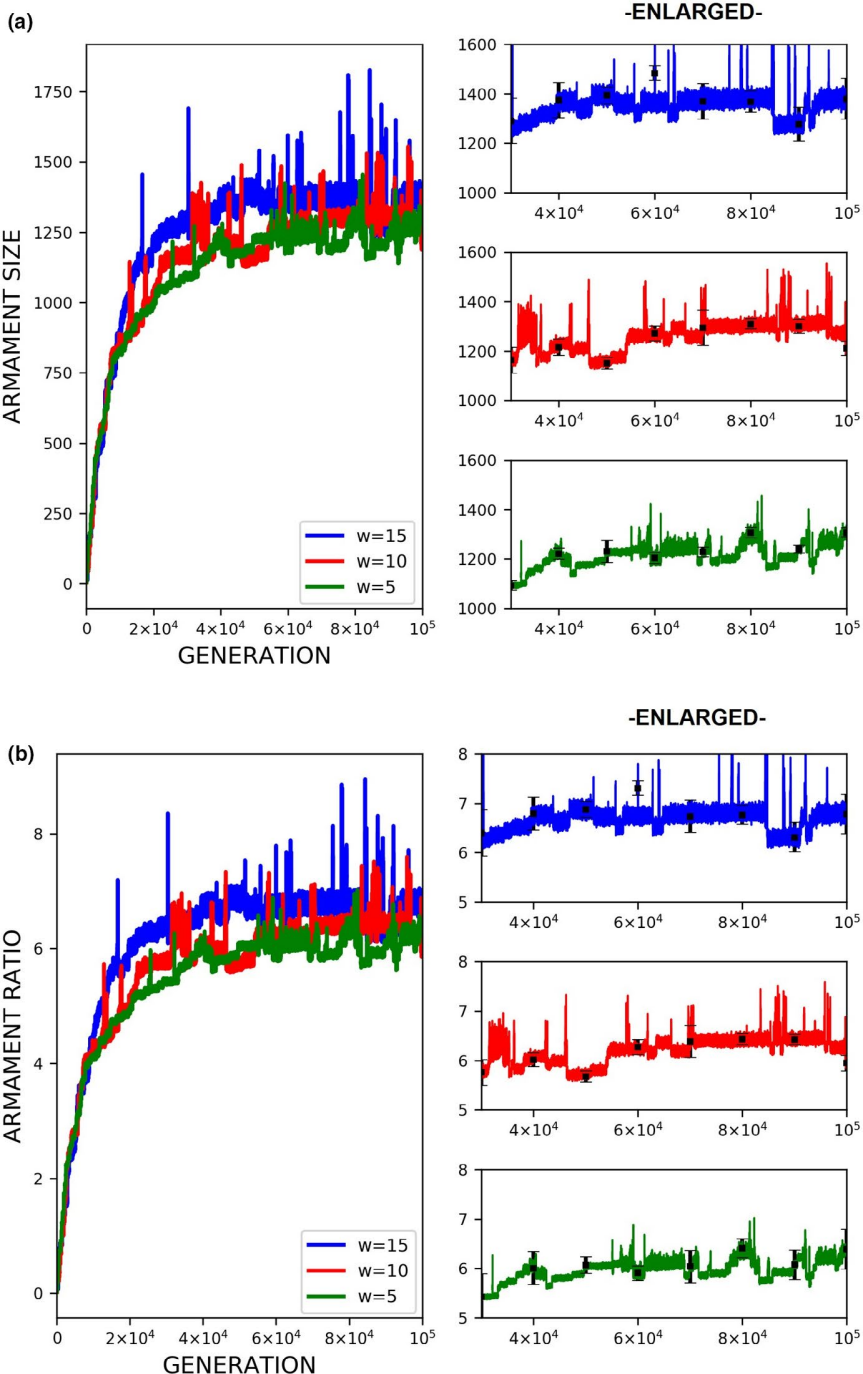
Evolution of armament size as influenced by competition cost. Enlarged diagrams show the evolution of (a) armament size and (b) armament ratio for generations *t*∈[40000,100000] as influenced by the competition cost. Parameters (see Table 1): *n*_1_=100, *n*_2_=100, *k* = 1.35, *b* ~ *N*(200,15), where *b*∈ [*b_min_*=150_,_ *b_max_*=250], *N* = 100,000, *r_thres_* =1.5, *W* = 15, *L* = 30 (blue), *W* = 10, *L* = 20 (red), *W* = 5, *L* = 10 (green), and *m*(*a_rt_*)= $\frac{1}{9^{10}}{\left(a_{\mathrm{rt}}‐1\right)}^{10}$. For our simulation analysis, we used the package Spyder (Python 3.7). Average and standard deviation of 20 runs for 100,000 generations are shown

**FIGURE 7:**
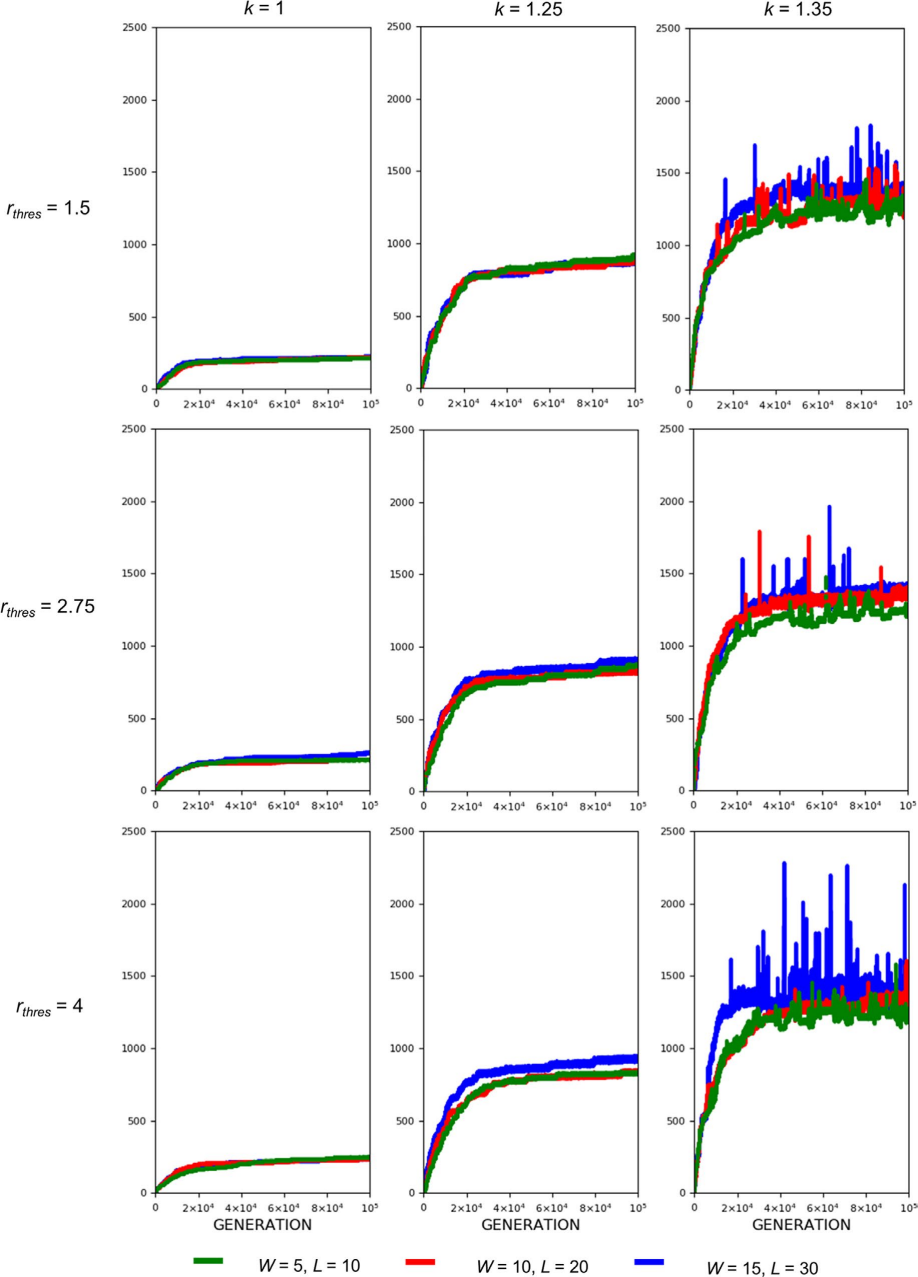
Evolution of armament size as influenced by competition cost. Competition costs are set as follows: *W* = 15, *L* = 30 (blue), *W* = 10, *L* = 20 (red), and *W* = 5, *L* = 10 (green). Parameters (see Table 1): *n*_1_=100, *n*_2_=100, *b* ~ *N*(200,15), where *b*∈[*b_min_*=150_,_ *b_max_*=250], *N* = 100,000, *r_thres_*∈{1.5,2.75,4}, *k*∈{1,1.25,1.35}, and *m*(*a_rt_*)= $\frac{1}{9^{10}}{\left(a_{\mathrm{rt}}‐1\right)}^{10}$. For our simulation analysis, we used the package Spyder (Python 3.7). Average of 10 runs for 100,000 generations is shown

**FIGURE 8:**
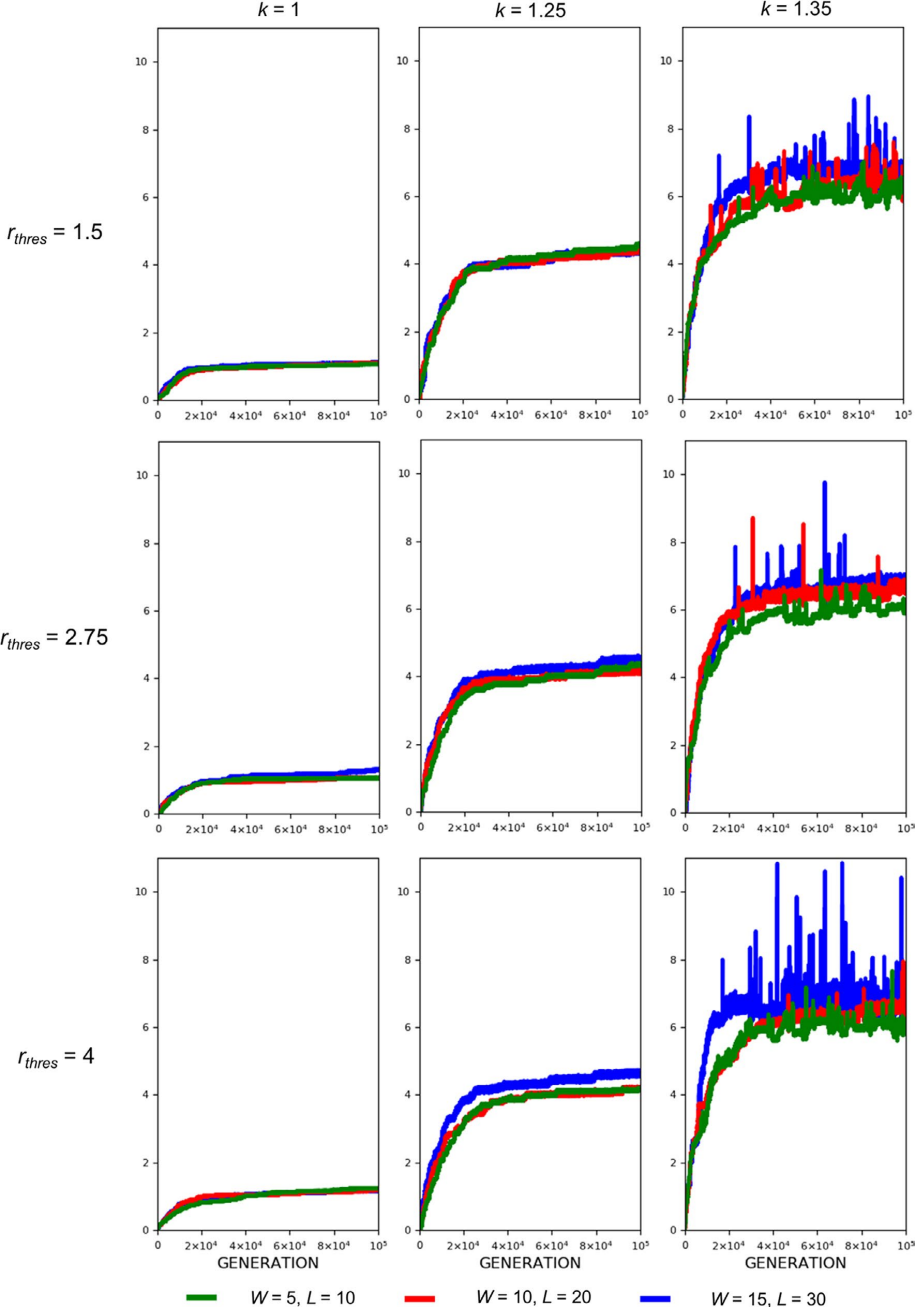
Evolution of armament ratio as influenced by competition cost. Competition costs are set as follows: *W* = 15, *L* = 30 (blue), *W* = 10, *L* = 20 (red), and *W* = 5, *L* = 10 (green). Parameters (see Table 1): *n*_1_=100, *n*_2_=100, *b* ~ *N*(200,15), where *b*∈[*b_min_*=150_,_ *b_max_*=250], *N* = 100,000, *r_thres_*∈{1.5,2.75,4}, *k*∈{1,1.25,1.35}, and *m*(*a_rt_*)= $\frac{1}{9^{10}}{\left(a_{\mathrm{rt}}‐1\right)}^{10}$. For our simulation analysis, we used the package Spyder (Python 3.7). Average of 10 runs for 100,000 generations is shown

Fights entailing high competition costs result in a slightly larger armament size and a higher armament ratio than fights entailing low competition costs when allometric slope k is high (Figure 6). However, this is not always the case for smaller values of k (Figures 7 and 8). In the previous simulations, we set a fixed ratio of *W* and *L*, that is, *L*=2*W*. We also looked at the effect of changing this ratio to the final armament size, that is, we fixed *W* = 5 and set *L* to 10, 15, 20, 25, and 30 (Figure 5). This is repeated for different values of k. There are no apparent differences in armament ratio and sizes when the ratio W and L are varied. This happens for different values of k.

**FIGURE 9:**
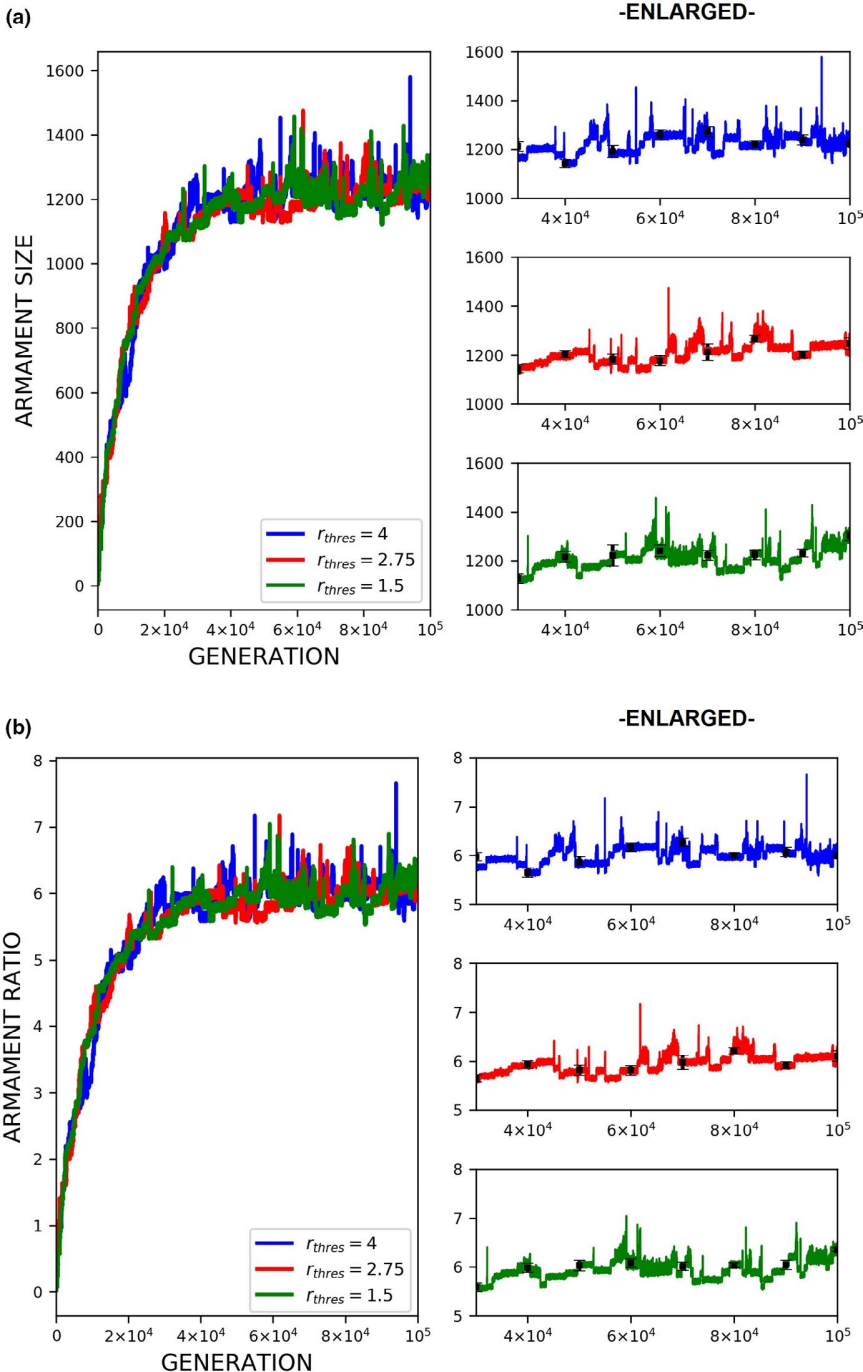
Evolution of armament size as influenced by the threshold ratio of the two armaments. Enlarged diagrams show the evolution of (a) armament size and (b) armament ratio for generations *t*∈[40000,100000] as influenced by the threshold ratio of two armaments. The nonlinearity of body size was set as follows: *r_thres_*=4 (blue), *r_thres_* =2.75 (red), and *r_thres_* =1.5 (green). Parameter values (see Table 1): *n*_1_=100, *n*_2_=100, *N* = 100,000, *f _i_*(1)=100, *b* ~ *N*(200,15), where *b*∈[*b_min_*=150_,_ *b_max_*=250], *W* = 5, *L* = 10, *m*(*a_rt_*)= $\frac{1}{9^{10}}{\left(a_{\mathrm{rt}}‐1\right)}^{10}$, and *k* = 1.35. For our simulation analysis, we used the package Spyder (Python 3.7). Average and standard deviation of 20 runs for 100,000 generations are shown

**FIGURE 10:**
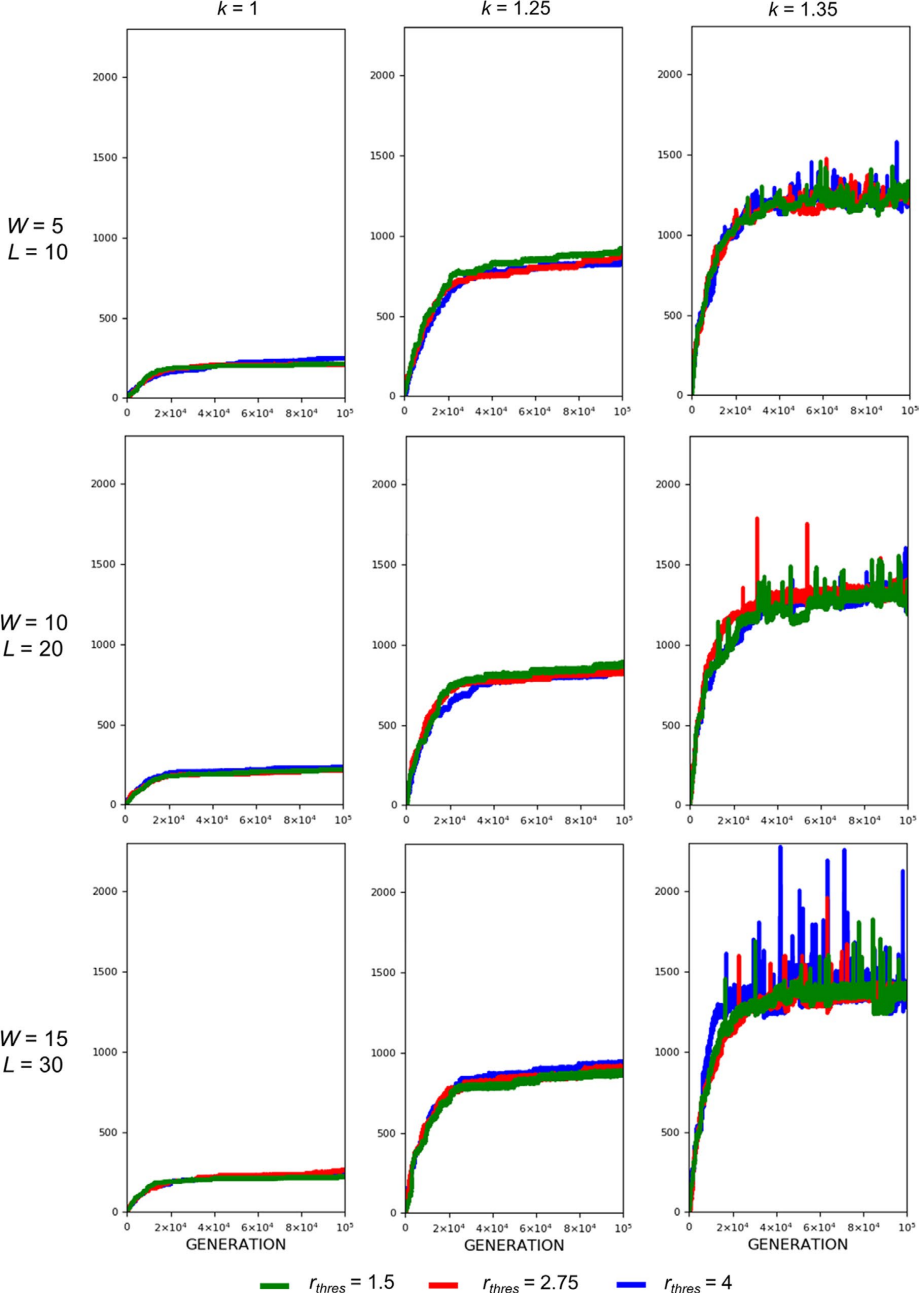
Evolution of armament size as influenced by the threshold ratio of the two armaments. The threshold ratios are as follows: *r_thres_*=4 (blue), *r_thres_* =2.75 (red), and *r_thres_* =1.5 (green). Parameter values (see Table 1): *n*_1_=100, *n*_2_=100, *N* = 100,000, *f _i_*(1)=100, *b* ~ *N*(200,15), where *b*∈[*b_min_*=150_,_ *b_max_*=250], *W*∈{5,10,15}, *m*(*a_rt_*)= $\frac{1}{9^{10}}{\left(a_{\mathrm{rt}}‐1\right)}^{10}$, and *k*∈{1,1.25,1.35}. For our simulation analysis, we used the package Spyder (Python 3.7). Average of 10 runs for 100,000 generations is shown

**FIGURE 11:**
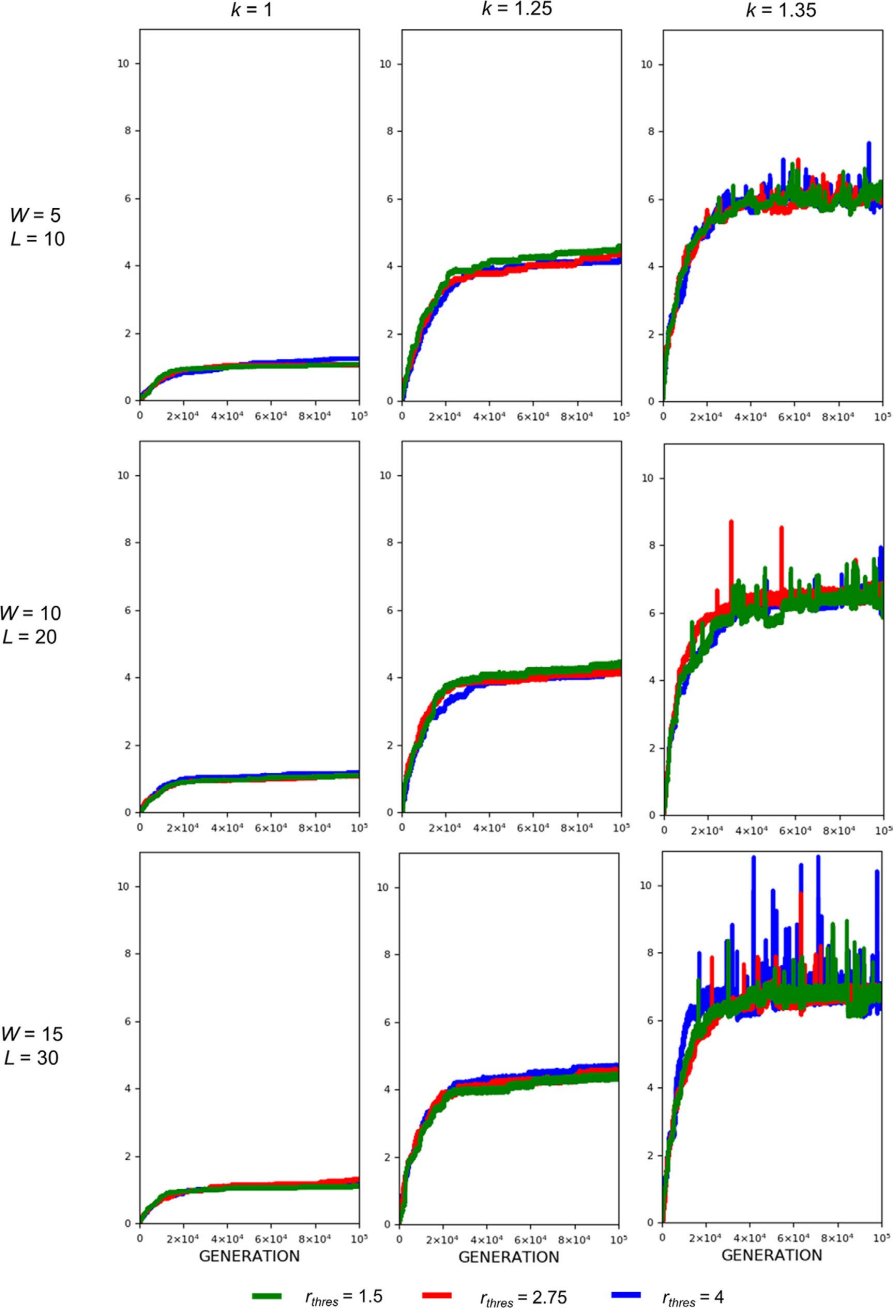
Evolution of armament ratio as influenced by the threshold ratio of the two armaments. The threshold ratios are as follows: *r_thres_*=4 (blue), *r_thres_* =2.75 (red), and *r_thres_* =1.5 (green). Parameter values (see Table 1): *n*_1_=100, *n*_2_=100, *N* = 100,000, *f_i_*(1)=100, *b* ~ *N*(200,15), where *b*∈[*b_min_*=150_,_ *b_max_*=250], *W*∈{5,10,15}, *m*(*a_rt_*)= $\frac{1}{9^{10}}{\left(a_{\mathrm{rt}}‐1\right)}^{10}$, and *k*∈{1,1.25,1.35}. For our simulation analysis, we used the package Spyder (Python 3.7). Average of 10 runs for 100,000 generations is shown

We also looked at the effect of threshold ratio *r_thres_*. For any value of the threshold ratio, armament will evolve exaggeratedly (Figure 9, *k* = 1.35). From Figures 10 and 11, it can be observed that the plots for the evolution of armament coincide at different values of *r_thres_*, when *k* is 1.35. This is evident for all competition costs used in this paper.

**FIGURE 12:**
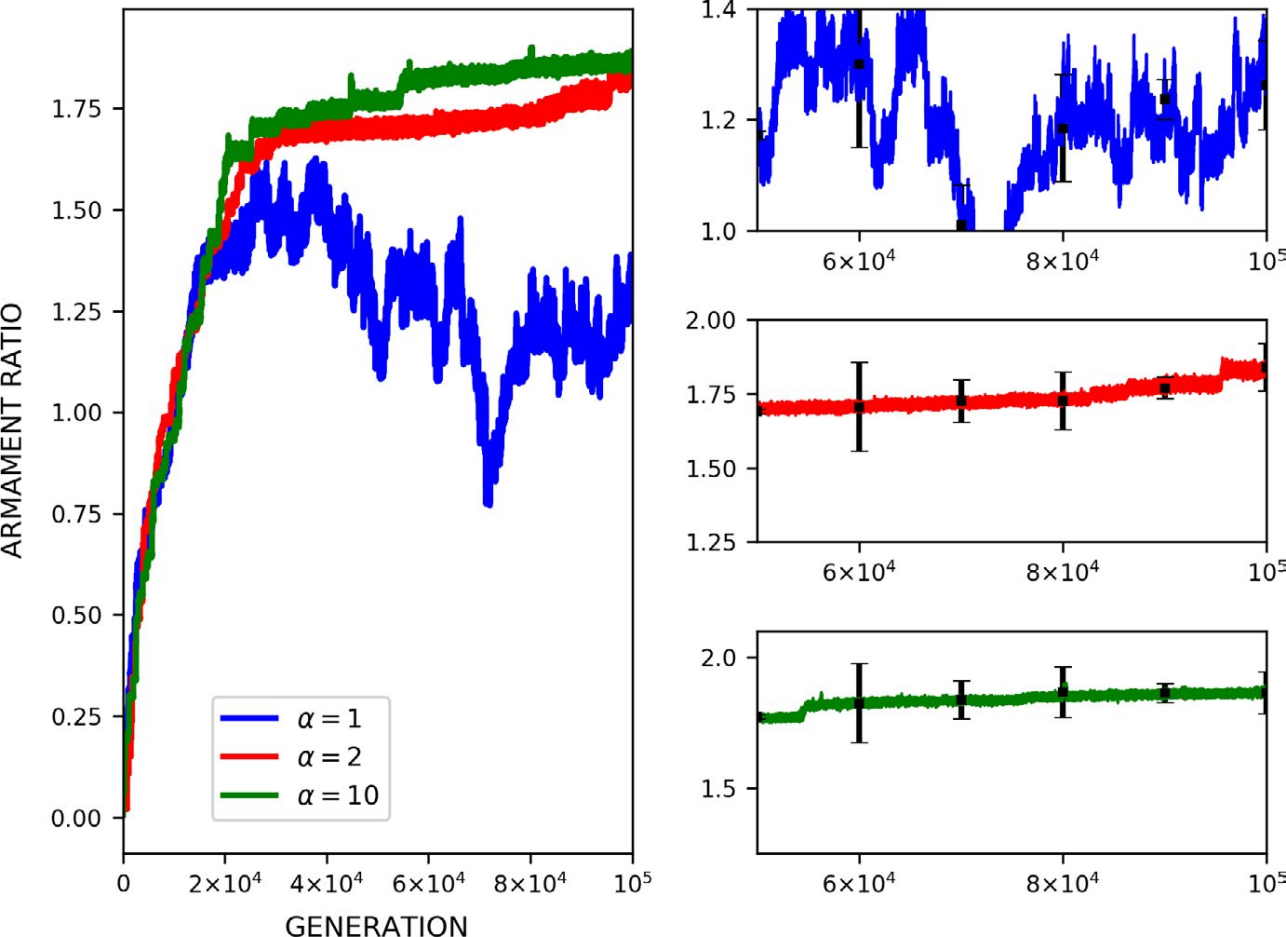
Effect of mortality rate function in the evolution of armament ratio. Mortality rate function *m* (Equation 4) is as follows: *m*(*a_rt_*)= $\frac{1}{9}\left(a_{\mathrm{rt}}‐1\right)$ (blue), *m*(*a_rt_*)= $\frac{1}{9^{2}}{\left(a_{\mathrm{rt}}‐1\right)}^{2}$ (red), and *m*(*a_rt_*)= $\frac{1}{9^{10}}{\left(a_{\mathrm{rt}}‐1\right)}^{10}$ (green). Parameter values (see Table 1): *n*_1_=100, *n*_2_=100, *N* = 100,000, *f_i_*(1)=100, *b* ~ *N*(200,15), where *b*∈ [*b_min_*=150_,_ *b_max_*=250], *W* = 5, *L* = 10, *r_thres_*=1.5, and *k = *1.35. For our simulation analysis, we used the package Spyder (Python 3.7). Average and standard deviation of 10 runs for 100,000 generations are shown

Our results show that armaments evolve exaggeratedly via male–male competition when there is a strong allometric relationship. Moreover, high competition costs will further the armament size and ratio in the presence of a strong allometric relationship. Take note that the mortality rate also has a profound effect on the armament ratio specifically when the exaggerated armament size can be several times larger than the body size. The resulting armament ratio depends on the given mortality rate function (Figure 12), indicating that the armament ratio (or size) increasingly evolves almost indefinitely unless mortality overcomes the advantage of mate competition.

## 3 DISCUSSIONS

The exaggerated evolution hypothesis offers a resolution to the question of how and when an exaggerated trait evolves in an animal. The hypothesis is that the rate of evolution of armament is escalated by male–male competition. Intrasexual selection is an outcome of the male–male competition (Berglund et al., 1996). It is advantageous for males to have a superior physical condition to obtain at least one mate (Kodrid‐Brown et al., 2006). Male armaments, as weapons during a competition, have evolved to increase the mating success of the bearer in a competitive environment. Different species have different physical armaments and behavioral traits, for example, teeth, horns, claws, aggression, and physical strength. Males with poor‐quality armaments in a population have a higher probability of reduced reproductive output, while winners have a higher probability of survival. However, the development of an armament is very costly (Tomkins et al., 2004). This study is the first simulation model that describes the evolution of armament size due to male–male competition. The aim of this research was to create a mathematical simulation model that gives insight into the development of male armament based on body size (allometry), which has never been done before.

In the present simulations, the mortality rate increases with the armament ratio (Figure 12). Moreover, an increase in alpha means a lower mortality rate as armament ratio increases. Equation (4) determines the effect of the evolution of armament on the percentage of males who eventually die (Ditchkoff et al., 2001; Johnstone et al., 2009). Hence, the size of the secondary sexual trait cannot increase indefinitely (Figure 12). Because of this, the size of secondary sexual male traits cannot increase indefinitely. This asymptotic increase in armament size is due to the opposing effects of sexual and natural selection. Sexual selection operates differently from natural selection as sexual selection arises from the differential ability of individuals to acquire mates (Yoshimura, 1992). In sexual selection theory, sexual selection on an armament will be offset by natural selection on the armament such that an optimal armament size exists.

The current simulation experiments show that the exaggeration of armament size (or ratio) occurs as a result of male–male competition for mates, as predicted by Darwin. The paradox proposed by Darwin is the extreme exaggeration of animal horns, which appear to be highly maladaptive in terms of natural selection (Darwin, 1871). Darwin presented a resolution to this paradox in his sequel to *The Origin of Species*. Our results show that for allometric relationship *k*, higher values of *k* will give larger armament size and ratio for all combinations of competition costs and threshold ratio explored in the study. This means that a strong allometric relationship will result in a bigger armament size regardless of the injury obtained by both individuals in case of a fight happening. The latter is dependent on the ratio of the armament of competing individuals. This is also true for different ratios of competition costs, which shows that strong allometric relationships will result in bigger armament size regardless of the amount of injury inflicted by the winner to the loser (Eberhard et al., 2018; Tidiere et al., 2017).

Regarding competition costs *W* and *L,* the effect of setting high costs can be noticed when *k* is high (Figures 7 and 8). Results show slightly higher armament size and ratio when competition costs are both increased and if *k* = 1.35. This is because greater competition cost will cause the individuals with a low fighting ability (a function of armament size, power function of body size, and physical condition) to be replaced by individuals with relatively larger armament size, which happens when *k* is high. From Figure 5, no apparent pattern is observed when the injury attained by the winner is fixed (e.g., equal to 5) and that of the loser is varied. However, for larger values of *k, that is*, *k* = 1.3 and 1.35, the highest value of *L* will result in the largest armament size and ratio at *t* = 100,000.

For threshold ratio *r_thres_,* exaggerated armament is evident for large values of *k, that is*, *k* = 1.25 and 1.35 for any value of *r_thres_* (Figures 10 and 11). This was expected since there will always be a winner, which has the opportunity to reproduce whether or not a fight will take place. From the same figures, plots for the evolution of armament coincide at different values of *r*_*thres*_, when *k* is 1.35. This is because large oscillations are present as compared to other values of *k,* indicating that the mortality function is regulating the armament size.

Moreover, results show that the exaggeration of armaments is plausible if mortality does not immediately limit armament size (Figure 12). To examine this mortality effect on the final armament ratio, we test different types of mortality curves (Figure 12). When the mortality trade‐off is weak, the armament ratio (or size) is either increased or decreased depending on the functions used for mortality (Equation 4). For all mortality functions, the mortality rates for the stable armament ratios depend on the convexity of the mortality rate function (Figure 12). Male–male competition will promote the exaggeration of armament size until mortality (in the sense of natural selection) limits the enlargement of these armaments. A continuous and persistent increase in mortality (e.g., due to the climatic changes) may lead to the termination of armament evolution. This result is consistent with the empirical findings of Ditchkoff et al. (2001). In their study, they showed that the probability of mortality increased as the size of the armament increased.

Here, we simply demonstrate the escalation of the rate of evolution of armaments via male–male competition. Therefore, our simulation is simplified in many aspects. For example, we held the population size and numbers of males and females (100 individuals each) for each generation. We investigate the particular parameters that contributed to the exaggerated evolution of armaments, including allometric slope, competition cost, and the ratio of the two armaments. Armaments evolve according to several factors such as fighting ability and allometric slope (Figures 2, 3, 4; Eberhard et al., 2018; Pomfret & Knell, 2006; Tidiere et al., 2017); fighting ability and competition cost (Figures 5, 6, 7, 8; Johnstone et al., 2009; Lailvaux et al., 2005); and fighting ability and threshold ratio of the two armaments (Figures 9, 10, 11; Karino et al., 2005). Note that other factors that could limit the size of the armament are structural failure of oversized horn (McCullough, 2014), biomechanics (Voje, 2016), and the trade‐off between armament size and testis size (Simmons & Emlen, 2006). However, we only considered the effect of genetic and male mate competition in the rate of evolution of armaments.

We develop three predictions based on the current results: (a) Sexual selection leading to armament evolution is balanced by the increasing mortality incurred by the growing armament size in males, (b) if the mortality rate increases with near‐zero armament ratios (Figure 12), the armament may be very small, or not developed at all, and (c) exaggerated armaments can evolve if the mortality rate increases very slowly with the armament ratio (high convexity; Figure 12). The first two predictions may be observed in nature or be tested empirically in the future. The third prediction offers a potential resolution to the paradox proposed by Darwin. However, empirical testing of this prediction might be difficult because it involves the past evolutionary history of armament size.

Thus, our research, the mathematical simulation, is consistent with the models and empirical findings of Ditchkoff et al. (2001), Eberhard et al, (2018), Karino et al. (2005), Lailvaux et al. (2005), Pomfret and Knell (2006), Johnstone et al. (2009), and Tidiere et al. (2017). The result of this paper is an additional type of theoretical evidence to the exaggerated evolution of male armaments via male–male competition.

## CONFLICT OF INTEREST

The authors declare no conflict of interest.

## AUTHORS CONTRIBUTION

**Maica Krizna Areja Gavina:** Conceptualization (supporting); Formal analysis (equal); Methodology (equal); Project administration (lead); Writing‐original draft (lead); Writing‐review & editing (lead). **Monica Torres:** Methodology (equal); Software (equal); Writing‐original draft (equal); Writing‐review & editing (supporting). **Gimelle Gamilla:** Methodology (equal); Software (equal); Writing‐original draft (equal); Writing‐review & editing (supporting). **Tomohiko Sakaguchi:** Conceptualization (supporting); Methodology (supporting); Writing‐original draft (equal). **Hiromu Ito:** Methodology (equal); Writing‐original draft (equal). **Jomar F. Rabajante:** Formal analysis (equal); Writing‐original draft (equal); Writing‐review & editing (supporting). **Jerrold** **Maranan Tubay:** Formal analysis (equal); Writing‐original draft (equal); Writing‐review & editing (supporting). **Jin Yoshimura:** Conceptualization (lead); Formal analysis (equal); Methodology (equal); Writing‐original draft (equal). **Satoru Morita:** Conceptualization (supporting); Methodology (equal); Writing‐original draft (equal).

## Data Availability

The accompanying code is available on Data Dryad (https://doi.org/10.5061/dryad.wstqjq2kp).
